# Effect of body weight at photostimulation on productive performance and welfare aspects of commercial layers

**DOI:** 10.5713/ab.22.0365

**Published:** 2023-02-27

**Authors:** Fazal Raziq, Jibran Hussain, Sohail Ahmad, Muhammad Asif Hussain, Muhammad Tahir Khan, Assad Ullah, Muhammad Qumar, Fazal Wadood, Gull-e-Faran

**Affiliations:** 1 Department of Poultry Production, Faculty of Animal Production and Technology, University of Veterinary and Animal Sciences, Lahore-54000, Pakistan; 2 College of Animal Husbandry and Veterinary Sciences, Abdul Wali khan University, Mardan-23200, Pakistan; 3 Department of Poultry Science, Faculty of Animal Production and Technology, Cholistan University of Veterinary and Animal Sciences, Bahawalpur-63100, Pakistan; 4 Civil Veterinary Hospital Gumbat, Kohat-26120, Pakistan; 5 Department of Animal Nutrition, Faculty of Animal Production and Technology, Cholistan University of Veterinary and Animal Sciences, Bahawalpur-63100, Pakistan; 6 Department of Theriogenology, Faculty of Veterinary Science, Cholistan University of Veterinary and Animal Sciences, Bahawalpur-63100, Pakistan; 7 Department of Biochemistry, Institute of Biochemistry, Biotechnology and Bioinformatics, The Islamia University of Bahawalpur-63100, Pakistan

**Keywords:** Body Weight, Egg Quality, Hormonal Profile, Productive Performance, Welfare

## Abstract

**Objective:**

Due to current selection practices for increased egg production and peak persistency, the production profile, age at maturity, and body weight criteria for commercial layers are constantly changing. Body weight and age at the time of photostimulation will thus always be the factors that need to be adequately addressed among various production systems. The current study was carried out to determine the effects of pullets’ body weight (low, medium, and heavy) on their performance, welfare, physiological response, and hormonal profile.

**Methods:**

With regard to live weight, 150 16-week-old pullets were divided into three groups using a completely randomized design (CRD) and held until the 50th week. One-way analysis of variance was used to evaluate the data under the CRD, and the least significant difference test was used to distinguish between treatment means.

**Results:**

In comparison to the medium and light birds, the heavy birds had higher body weight at maturity, an earlier age at maturity, and higher egg weight, eggshell weight, eggshell thickness, egg yolk index, breaking strength, egg surface area, egg shape index, egg volume, and hormonal profile except corticosterone. However, the medium and light birds had lower feed consumption rates per dozen eggs and per kilogram of egg mass than the heavy birds. Light birds showed greater body weight gain, egg production, and egg specific gravity than the other categories. At 20 weeks of age, physiological response, welfare aspects, and catalase were non-significant; however, at 50 weeks of age, all these factors—aside from catalase—were extremely significant.

**Conclusion:**

The findings of this study indicate that layers can function at lower body weights during photostimulation; hence, dietary regimens that result in lighter pullets may be preferable. Additionally, the welfare of the birds was not compromised by the lighter weight group.

## INTRODUCTION

Due to the increasing need for animal origin proteins brought on by the rapid rise in global population, poultry has taken the lead in meeting this demand, and as a result, extensive research is being done to improve bird performance. The efficiency of egg production and the features of the eggs are influenced by the weight of the chickens at the beginning of lay and throughout the production year [1]. In addition to feed conversion ratio (FCR), body weight, which is a result of skeletal size, fleshing (muscle), and condition (fat), is an important metric to monitor throughout the life of the bird [2]. There is proof that a close relationship exists between body weight and the onset of lay, a sign of sexual maturity [3].

The photostimulation of laying birds at particular ages and weights has a significant impact on body weight gain and total production efficiency [4]. The first egg’s weight is affected by the birds’ weight and how they develop during the growth phase [5]. An increase in body weight in laying hens will reduce egg output while increasing egg weight and feed intake, according to earlier studies. The explanation is because heavier birds consume more feed and produce larger eggs than lighter birds [6]. The association between egg weight, mature body weight, and egg production has the same pattern as that shown in the body weight at sexual maturity [7]. Due to current selection practices for increased egg production and peak persistency, the production profile, age at maturity, and body weight criteria for commercial layers are constantly changing. Considering this, the current study was carried out to assess the effects of pullets’ body weight (low, medium, and heavy) on their performance, welfare, physiological response, and hormonal profile.

## MATERIALS AND METHODS

### Animal care

All experimental protocols were authorized by the University of Veterinary and Animal Sciences (UVAS), Lahore Ethical Review Committee via letter number DR/985 and followed all laws and regulations. The experiment ran from week 17 until week 50 (34 wks).

### Location and duration of experiment

The study was conducted at the Department of Poultry Production, UVAS, Ravi Campus, Pattoki. Pattoki is located at 31°1′ 0N and 73°50′ 60E with an altitude of 186 m (610 ft). This city experiences normally hot and humid tropical climate with temperature ranging from 05°C in winter and +45°C in summer.

### Experimental birds and husbandry

**Figure 1:**
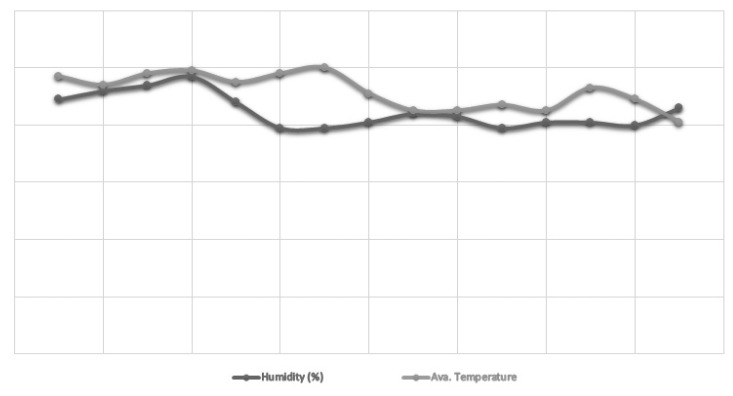
Variations in temperature (°F) and humidity (%) by a wet and dry bulb hygrometer (Mason’s type, Zeal, England).

In total, 150 commercial layer pullets (LSL lite) were procured from a flock that had already been raised at the Department of Poultry Production. Up to 15 weeks, this flock was kept under standard management practices. These pullets were divided into three groups at 16 weeks of age according to live weight using a completely randomized design and five replicates of ten birds each. At the time of photostimulation, the body weight categories used in this investigation were light (under 1,300 g), medium (1,300 to 1,375 g), and heavy (more than 1,375 g). The pullets were housed in independent open-sided laying house with east-west dimensions of 6.10× 6.10 m (37.21 m^2^) and 3-tier laying cages of 5.18×1.52 m (47.42 m^2^). To make the process of collecting eggs easier, the cages had a sloping wire floor. Ceiling fans, curtains, and other practical manual methods were used to regulate the ventilation, humidity, and interior temperature. A wet and dry bulb hygrometer (Mason’s type, Zeal, England) positioned in the middle of the home was used to record daily variations in temperature (°F) and relative humidity at 6:00 AM and 6:00 PM (Figure 1). To remove faeces, removable dropping trays were installed beneath the mesh floor. The birds were fed using detachable individual trough feeders that were installed outside the cage. At 6:00 AM, the birds were given a commercial laying diet (Table 1) with an allotment of 90 g/bird/d, and freshwater availability was guaranteed by the automatic nipple drinker system installed inside. The birds were given access to natural day light at the beginning of the experiment, and then the amount of light was increased by 30 minutes every week until a 16 L:8 D photoperiod was reached. The experiment lasted up to the age of 50 weeks.

### Light intensity

Throughout the phases of rearing and production, the birds were housed in open-sided house. The birds were kept at their natural day length for the whole growing season (up to 15 weeks) because the flock was in-season, placed in the middle of April. The growth period’s natural daytime length was roughly 12 hours. The photoperiod was extended for photostimulation by 30 minutes per week until a total day length of 16 hours was reached. In the production phase, the birds were exposed to 40 to 50 lux of light intensity after sunset.

### Data and sample collection

#### Production performance

Age and body weight at the first egg were noted at the onset of egg production. To compute the percent egg production and egg mass, the daily egg number and egg weight were also recorded. Also measured were FCR per dozen eggs and FCR per kilogram of egg mass. FCR per dozen eggs was measured in kilograms of feed consumed per dozen eggs produced whereas FCR per kg egg mass was expressed as kilograms of feed consumed per kilogram of egg produced.

#### Egg quality

Analysis of the egg quality was done at 20 and 50 weeks. Five eggs were taken for this purpose from each replicate. These eggs were evaluated for egg specific gravity estimate. The eggshell thickness of each egg was measured using a micrometer screw gauge. Albumen height of each egg was measured using Digital Haugh tester (ORKA Food Technology Ltd., Ramat Hasharon, Israel) and the measurement was used to calculate Haugh unit (HU) score using the formula HU = 100×log (H–1.7×W^0.37^+7.6) where H is the height of albumen (mm) and W is the egg weight (g). Yolk index was also measured as a ratio of yolk height to yolk width. Eggshell breaking strength (N) was also measured by placing the eggs lengthwise and using egg force reader (ORKA Food Technology Ltd, Ramat Hasharon, Israel). Data regarding egg geometry (egg surface area, egg shape index, and egg volume) were recorded.

#### Welfare data

Welfare characteristics footpad dermatitis (FPD) and feather condition (FC) were among the variables that were recorded. Individual birds were examined to determine the FC score. Each hen’s back feather coverage was graded on a six-point scale (0 to 5) depending on the state of its feathers, as follows: (0 = fully feathered hen, 1 = rough feathered hen, 2 = some broken feathers, 3 = extensively broken feathers, 4 = almost bald, and 5 = baldness [8].

Footpad dermatitis was graded on a three-point scale (1 to 3), with 1 denoting poor FPD condition with blood or sever lesions and 2 denoting average FPD condition with swollen lesions, respectively. A score of 3 denoted normal FPD condition with no abnormalities.

#### Physiological response

The physiological response of the birds was evaluated by monitoring the birds’ respiration rate, heartbeat rate and body temperature. Holding the birds inverted for a minute, we tracked the abdomen movements to determine the respiration rate. With the help of a stethoscope (3M Littmann Mater Classic II 1392; 3M Co., Ltd., St. Paul, MN, USA), the heart rate was determined. Using a digital translucent thermometer from Medicare (MANA & Co, Pattoki, Pakistan), the rectal temperature (°C) was recorded.

#### Hormonal profile

Blood samples (3 mL) were drawn from the wing brachial veins of 15 randomly chosen birds (three per replication) on weeks 20 and 50 at 8:00 in the morning. Blood samples were taken using monovette syringes that had been heparinized (50 IU/mL). After sampling, the birds were gently put back in their own cages without causing them any additional pain. The serum was then extracted from the blood samples after being centrifuged at 3,000 rpm for 15 minutes and sent to a private laboratory (Decent Hormone Lab, Lahore, Pakistan) for determination of triiodothyronine (T_3_) using total T3 RIA Kit (Ref # IM199 & IM3287), thyroxin (T_4_) using total T4 RIA Kit (Ref # IM1447 and IM3286), gonadotropin releasing hormone (GnRH) using Elabscience (Lot No # E1TF7MCWQB), follicle stimulating hormone (FSH) using FSH IRMA Kit (Ref # IM2125 and IM3301), corticosterone using RIA Kit (Ref # IM841), luteinizing hormone (LH) using LH IRMA Kit (Ref # IM1381 and IM3302) and catalase.

### Statistical analysis

Analysis of collected data was performed using one-way analysis of variance (ANOVA) in SAS 9.1. Significant treatment means were separated through least significant difference test.

The statistical model used was:


$${\text{Y}}_{\text{ij}}=\mu +{\text{t}}_{\text{i}}+\Sigma \text{ij}$$

Where; Y_ij_ = observation of dependent variable documented on ith treatment; μ = Population mean; t_i_ = Effect of ith treatment i.e. body weight at photostimulation (i = 1, 2, 3); Σij = Residual outcome of jthobservation in ith treatment NID ~ 0, σ^2^.

## RESULTS

### Effect of body weight groups on production performance

The results showed that the heavy birds reached sexual maturity (age at first egg) earlier than the light and medium birds, whereas the light birds produced more eggs overall, gained more body weight, and had better FCR per dozen eggs as well as per kilogram of egg mass. However, compared to the medium and light birds, the heavy demonstrated higher egg mass (Table 2).

### Effect of body weight groups on egg quality traits

At 20 weeks of age, body weight categories had no effect on the characteristics of eggs that determine their quality, except for egg weight, eggshell weight, eggshell thickness, egg yolk index, and HU. However, at 50 weeks of age, significant effects of body weight categories on egg quality were observed (Table 3).

### Effect of body weight groups on egg geometry

Egg geometry characteristics, except for egg shape index, did not differ significantly among body weight groups at 20 weeks of age, according to an ANOVA, but at 50 weeks of age, those differences became significant (Table 4).

### Effect of body weight groups on welfare traits

At 20 weeks of age, there were no statistically significant differences between the groups in terms of bird welfare aspects such FPD and FC. However, compared to the medium and light birds at 50 weeks of age, the heavier birds had poorer footpad and better FC (Table 5).

### Effect of body weight groups on physiological response

At 20 weeks of age, the physiological responses of the birds were not significantly different for body temperature, heart rate, and respiration rate depending on body weight categories, but at 50 weeks of age, the light birds had higher values for rectal temperature, heart rate, and respiratory rate than the medium and heavy birds (Table 6).

### Effect of body weight groups on hormonal profile

Except for catalase, body weight categories had a strong impact on both productive and reproductive hormones (Table 7). At 20 and 50 weeks of age, heavier birds than medium and light birds displayed higher levels of triiodothyronine (T_3_), thyroxine (T_4_), GnRH, FSH, and LH. However, light birds had the greatest corticosterone levels, followed by medium and large birds (Table 7).

## DISCUSSION

### Effect of body weight groups on production performance

Cost considerations are important in the business of raising livestock, especially poultry. For instance, between 65% and 70% of the overall cost of growing poultry is attributable to feed costs. Poultry body weight plays a key role in feed intake, feed efficiency and other egg characteristics [9]. Similar to our findings, research has shown that heavier relative to lighter body weight layers consume more feed [10]. This change may be the result of increased body maintenance requirements.

There are many elements that influence the economics of chicken production, but two of them—faster growth and effective feed conversion—play a significant part in the economics of the poultry industry. Lacin et al [9]’s observation that the body weight of the laying hens considerably impacts the FCR corroborated the relationship between the FCR and live body weight in the current research.

### Effect of body weight groups on egg quality traits

When it comes to poultry birds, the age at sexual maturity is crucial. According to Pishnamazi et al [11], the only factor that affected differences in body conformation at sexual maturity was body weight. In comparison to lighter-weight poultry birds, heavier-weight birds reach sexual maturity sooner [12]. The plasma estradiol-17β content may be the cause of this variance. According to Triyuwanta et al [13], variations in egg production and egg weight have been linked to variations in the body weight of layers during sexual maturity. It has been noted that heavier relative to lighter weight layers produce more eggs [14]. The plasma estradiol-17β content may be the cause of this variance.

Romero et al [15], who noted a positive correlation between hen body weight and its egg weight, support the positive association between body weight and egg weight in this research. It has been noted that heavier body weight layers tend to lay heavier eggs than lighter weight layers [10]. This difference may be brought on by growing ova and secreting more albumen. Specific gravity is a sign of the quality and freshness of the eggshell because eggs with a stronger shell have a higher specific gravity than eggs with a thin shell. Eggs that have just been laid have a higher specific gravity than long and old stored eggs. In the current investigation, it was discovered that layers with lighter body weights had eggs with a higher specific gravity. Similar to this, it has been noted that layers with greater body weights have lower egg specific gravities than those with lighter body weights [16]. The relationship between increased body weight and eggs decreased specific gravity may be to blame. Mekky et al [17]’s conclusion that pullets with larger body weight laid eggs with increased eggshell weight and eggshell thickness as compared to layers with lighter body weight supports our findings regarding eggshell weight. This could be explained by the increased calcium deposition in eggshells caused by heavier body weight, which results in higher egg weight.

### Effect of body weight groups on egg geometry

The calculation of egg geometry is crucial to the poultry business. It has been noted that strains with heavier body weights tend to lay eggs with greater egg length and width [18]. The egg geometry has been shown to be significantly influenced by body weight [9]. It has been noted that layers with lower body weight than medium and overweight have a lowered egg shape index [19]. This change may be brought on by layers’ advancing age and weight. In a similar manner, larger egg weight may have contributed to the overweight group’s highest values for egg volume and surface area.

### Effect of body weight groups on welfare traits

Footpad dermatitis is a financial and welfare indicator for poultry. Among layers that have been produced commercially, FPD is a frequent and complicated problem. Weight of the bird is one of the causes of FPD [20]. When compared to other body weight groups in the current investigation, FPD was considerably higher in the overweight birds. Shepherd and Fairchild [21], who noted a severe FPD incidence in broiler chicks with high body weight, provide support for these findings. In a similar vein, Hocking and Wu [22] found that FPD was higher in layers with heavier body weights compared to layers with medium and lighter body weights, demonstrating a substantial correlation between FPD and body weight.

For the welfare of hens and as an economic production criterion, feather score is crucial. In the current study, bigger body weight layers exhibited improved FC compared to medium and lighter body weight layers. These findings are further corroborated by Kiani and von Borstel [23], who found that larger laying hens had superior back FC than lighter and medium-weight hens. This might be explained by heavier bodyweight layers producing more eggs and having higher plasma estradiol-17 concentrations [4].

### Effect of body weight groups on physiological response

Human literature has long documented the consequences of a high body weight, such as that caused by obesity, on respiratory and physical functioning as well as the vascular system. Large-bodied, productive laying birds were found to have a higher respiratory rate. This accelerated respiration may be a compensatory response to metabolic acidosis during the production of eggshell CaCO3 [24]. In the present trial, a highest body temperature was recorded in light birds. Similar to this, Ghayas et al [25] found that lighter broilers had higher rectal temperatures than heavier broilers. This can be the outcome of elevated T_3_, which might have contributed to elevated heart rate in layer.

### Effect of body weight groups on hormonal profile

Triiodothyronine (T3), a plasma thyroid hormone, is important for promoting broiler growth and for accelerating the compensatory growth of birds. Hassaan et al [26]’s revelation of a higher T_3_ level in heavier hens as compared to lighter hens supports the current findings. This might be caused by variations in the hormonal composition or metabolic processes between lines. The current study’s heavier birds had greater FSH and LH values at 20 and 50 weeks of age than the medium and light birds. Similar to the current findings, greater FSH and LH levels upon phostimulation have been seen in heavier compared to medium and lesser body weight broiler breeder groups [27]. This variation might be due to body mass, because FSH helps in fat accumulation. The main glucocorticoid secreted in poultry chickens under stress is corticosterone (a stress hormone, in all vertebrate classes); it is the byproduct of the activation of the hypothalamic-pituitary-internal axis and has long-term effects on stress adaption. According to Huff et al [28], varied body weights have different levels of corticosterone, such as heavier and lighter poultry birds. In the current investigation, lighter body weight layers had higher serum corticosterone concentrations than other body categories. Similar to this, it has been noted that birds with lighter body weights have higher corticosterone levels than those with bigger body weights [29]. The metabolic rate may be the cause of this discrepancy.

Hydrogen peroxide (H2O2) is broken into water and oxygen by the enzyme catalase, which is present almost universally in all living things, exposed to oxygen and prevents oxidative cell damage. In the current research, birds with higher bulk had the highest catalase concentrations. Jena et al [30] findings’ that catalase levels are greater in birds with heavier body weights than in birds with lesser body weights are consistent with these findings since catalase concentration rises with age and body weight.

## CONCLUSION

Based on the current research, it may be deduced that layers can function at photostimulation with a lighter body weight; as a result, nutritional strategies that result in lighter pullets may be preferable. Additionally, the welfare of the birds was not compromised by the lighter weight group.

## Figures and Tables

**Table 1:** Composition of the ration offered to the experimental birds

Items	Quantity
Ingredient (%)	
Corn	62.30
Guar meal	3.00
Raw rice bran	4.00
Soybean meal 44%	1.31
Rape seed meal	2.00
DL-Methionine	0.23
L-threonine	0.08
Calcium carbonate	8.29
Salt	0.11
Corn gluten	1.00
Canola meal	8.00
Cotton seed meal	4.00
Lysine sulphate	0.36
Premix^1)^	0.30
L-Tryptophan	0.01
Fish meal 47%	1.00
Feather meal 54%	4.00
Quantum 600 FTU	0.01
Total	100.00
Nutrient (%)	
Crude protein	16.5
Metabolizable energy (kcal/kg)	2902
Calcium	3.55
Phosphorous	0.66
Sodium	0.16
Potassium	0.61
Lysine	0.82
Methionine	0.41

1) Provided per kg of diet: vitamin A, 11,000 IU; vitamin D_3_, 2,560 IU; vitamin E, 44 IU; vitamin K, 4.2 mg; riboflavin, 8.5 mg; niacin, 48.5 mg; thiamine, 3.5 mg; d-pantothenic, 27 mg; choline, 150 mg; vitamin B_12_, 33 μg; copper, 8 mg; zinc, 75 mg; manganese, 55 mg; iodine, 0.35 mg; selenium, 0.15 mg.

**Table 2:** Effect of body weight categories on production performance of commercial layers

Parameters	Body weight			

	Light	Medium	Heavy	p-value
AM (d)	142.00±2.35^a^	135.60±2.52^b^	131.60±1.44^c^	0.0002
WM (g)	1,264.00±10.77^c^	1,334.80±11.47^b^	1,402.00±15.08^a^	<0.0001
EN	198.44±2.49^a^	176.96±5.54^b^	180.66±1.51^b^	0.0016
EP (%)	81.06±1.02^a^	78.09±0.62^b^	79.23±2.26^b^	0.0018
EM (g)	8,499.78±113.35^b^	9,817.63±104.08^b^	10,150.57±51.37^a^	0.0027
FCR_DE_	1.56±0.02^b^	1.50±0.02^b^	1.77±0.06^a^	0.0035
FCR_EM_	2.50±0.04^b^	2.24±0.02^b^	3.05±0.08^a^	<0.0001
BWG (g)	417.50±3.23^a^	390.00±3.54^b^	374.00±2.92^c^	<0.0001

AM, age at maturity; WM, weight at maturity; EN, egg number; EP, egg production; EM, egg mass; FCR_DE_, feed conversion ratio per dozen eggs; FCR_EM_, feed conversion ratio per kg egg mass; BWG, body weight gain.

a–c Means within a row with different superscripts differ (p≤0.05).

**Table 3:** Effect of body weight categories on egg quality traits of commercial layers

Parameters	Body weight			

	Light	Medium	Heavy	p-value
Egg quality at 20 week age				
EW (g)	41.70±0.42^b^	42.64±0.26^b^	43.64±0.42^a^	0.0004
ESG	1.10±0.00	1.09±0.00	1.08±0.00	0.7022
EBS (N)	46.55±3.36	44.44±0.96	42.19±0.68	0.1851
EST (mm)	0.43±0.01^a^	0.38±0.01^b^	0.38±0.01^b^	0.0007
ESW (g)	7.12±0.04^c^	7.32±0.02^b^	7.42±0.02^a^	<0.0001
HU	91.87±2.10^a^	90.79±1.10^b^	84.82±1.31^c^	0.0001
YI (%)	46.43±0.74^a^	45.30±0.25^a^	37.22±0.20^b^	<0.0001
Egg quality at 50 week age				
EW (g)	51.28±0.29^c^	58.20±0.33^b^	62.82±0.44^a^	<0.0001
ESG	1.09±0.01^a^	1.08±0.01^b^	1.08±0.02^b^	0.0006
EBS(N)	36.58±1.22	37.93±2.20	40.46±0.76	0.2262
EST (mm)	0.38±0.01^b^	0.37±0.01^b^	0.40±0.01^a^	0.0003
ESW (g)	7.24±0.02^c^	7.46±0.02^b^	7.56±0.02^a^	<0.0001
HU	91.77±0.73^a^	89.75±1.14^b^	88.80±0.39^b^	0.0122
YI (%)	34.63±0.37^c^	40.06±0.52^b^	44.42±0.65^a^	<0.0001

EW, egg weight; ESG, egg specific gravity; EBS, egg breaking strength; EST, eggshell thickness; ESW, eggshell weight; HU, Haugh unit; YI, yolk index.

a–c Means within a row with different superscripts differ (p≤0.05).

**Table 4:** Effect of body weight categories on egg geometry of commercial layers

Parameters	Body weight			

	Light	Medium	Heavy	p-value
Egg geometry at 20 week age				
ESA (cm^2^)	55.80±0.82^b^	56.70±2.09^b^	57.53±1.01^a^	0.0056
ESI (%)	74.34±1.94	75.65±2.43	79.03±2.79	0.3951
EV(cm^3^)	38.40±0.59^b^	39.26±1.61^b^	40.17±0.70^a^	0.0053
Egg geometry at 50 week age				
ESA (cm^2^)	63.51±1.67^b^	69.23±1.09^b^	73.20±2.97^a^	0.0006
ESI (%)	72.74±0.79^b^	74.52±0.27^ab^	75.82±1.38^a^	0.0723
EV (cm^3^)	46.38±1.81^b^	53.10±1.14^b^	57.52±3.39^a^	0.0007

ESA, egg surface area; ESI, egg shape index; EV, egg volume.

a,b Means within a row with different superscripts differ (p≤0.05).

**Table 5:** Effect of body weight categories on welfare aspects of commercial layers

Parameters	Body weight			

	Light	Medium	Heavy	p-value
Welfare aspects at 20 week age				
FPD^1)^	0.20±0.20	0.20±0.20	0.40±0.40	0.7564
FC^2)^	0.40±0.24	0.20±0.20	0.60±0.24	0.4933
Welfare aspects at 50 week age				
FPD	1.40±0.24^b^	2.40±0.24^a^	3.20±0.37^a^	0.0035
FC	1.40±0.24^b^	2.40±0.37^a^	2.80±0.37^a^	0.0379

FPD, foot pad dermatitis; FC, feather condition.

1) FPD, 0 = no lesions; 4 = severe lesions.

2) FC, 0 = clean bird; 1 = slightly dirty feathers; 2 = very noticeably dirty bird; 3 = completely dirty bird.

a,b Means within a row with different superscripts differ (p≤0.05).

**Table 6:** Effect of body weight categories on physiological response of commercial layers

Parameters	Body weight			

	Light	Medium	Heavy	p-value
Physiological response at 20 week age				
RT (°C)	40.33±0.28	40.74.33±0.31	40.61±0.19	0.4184
HBR (beat/min)	271.30±4.51	270.30±4.65	284.80±0.40	0.1292
RR (breath/min)	19.40±0.81	20.80±0.42	19.30±0.87	0.2796
Physiological response at 50 week age				
RT (°C)	39.7860±0.24^a^	38.89±0.32^b^	38.33±0.32^c^	0.0002
HBR (beat/min)	314.00±7.48^a^	273.00±5.39^b^	258.80±3.31^b^	<0.0001
RR (breath/min)	22.80±0.37^a^	18.60±0.40^b^	17.20±0.37^c^	<0.0001

RT, rectal temperature; HBR, heartbeat rate; RR, respiratory rate.

a–c Means within a row with different superscripts differ (p≤0.05).

**Table 7:** Effect of body weight categories on hormonal profile of commercial layers

Parameters	Body weight			

	Light	Medium	Heavy	p-value
Hormonal profile at 20 week age				
T_3_ (nmol/L)	1.22±0.04^c^	1.41±0.02^b^	1.56±0.04^a^	<0.0001
T_4_ (nmol/L)	9.60±0.53^c^	13.15±0.45^b^	19.98±0.87^a^	<0.0001
GnRH (pg/mL)	58.77±4.78^c^	132.91±3.27^b^	199.75±15.58^a^	<0.0001
FSH (mIU/mL)	0.09±0.02^b^	0.18±0.02^b^	0.28±0.05^a^	0.0042
LH (mIU/mL)	0.04±0.00^c^	0.06±0.00^b^	0.08±0.00^a^	<0.0001
Cort (Nm)	17.28±0.33^a^	13.91±0.38^b^	10.99±0.46^c^	<0.0001
Cata (mM)	1.27±0.10	1.41±0.00	1.69±0.03	0.1875
Hormonal profile at 50 week age				
T_3_ (nmol/L)	1.37±0.03^c^	1.57±0.04^b^	1.86±0.04^a^	<0.0001
T_4_ (nmol/L)	11.48±0.38^c^	15.00±0.63^b^	25.06±0.84^a^	<0.0001
GnRH (pg/mL)	65.09±3.22^c^	146.90±2.36^b^	199.63±14.92^a^	<0.0001
FSH (mIU/mL)	0.15±0.05^c^	0.41±0.02^b^	0.60±0.05^a^	<0.0001
LH (mIU/mL)	0.06±0.01^b^	0.09±0.00^b^	1.08±0.30^a^	0.0018
Cort (Nm)	19.42±0.41^a^	15.80±0.42^b^	14.43±0.28^c^	<0.0001
Cata (mM)	1.44±0.07	1.64±0.04	1.90±0.00	0.0620

T_3_, triiodothyronine; T_4_, thyroxine; GnRH, gonadotropin releasing hormone; FSH, follicular stimulating hormone; LH, luteinizing hormone; Cort, corticosterone; Cata, catalase.

a–c Means within a row with different superscripts differ (p≤0.05).
